# Comprehensive analysis of miRNA expression, secretion profile, and functional network during myogenesis in response to spaceflight

**DOI:** 10.1038/s41526-026-00632-x

**Published:** 2026-07-11

**Authors:** Wenqing Kong, Mei Ma, Zhuoyang Li, Linjun Wang, Ruisen Ma, Siyi Shen, Xiaojun Yan, Ping Fan, Zhoumin Niu, Jingjing Chen, Tianyuan Qiu, Nuo Shi, Yuting Wu, Tao Zhang, Yuying Li, Hao Ying

**Affiliations:** 1 Shanghai Institute of Nutrition and Health, University of Chinese Academy of Sciences, Chinese Academy of Sciences and Shanghai Jiao Tong University Affiliated Sixth People’s Hospital, Shanghai, China; 2 Innovation Center for Intervention of Chronic Disease and Promotion of Health, Shanghai, China; 3 Shanghai Institute of Technical Physics of the Chinese Academy of Sciences, Shanghai, China; 4 Key Laboratory of Food Safety Risk Assessment, Ministry of Health, Beijing, China

**Keywords:** Cell biology, Diseases, Genetics, Molecular biology, Physiology

## Abstract

MicroRNAs (miRNAs) are key regulators of skeletal muscle development and homeostasis. As secretory factors, previous studies have identified and validated a space flight-associated circulating microRNA (miRNA) signature in response to space flight. However, the muscle-derived miRNAs are not clear. Here, we comprehensively analyzed the variations of miRNA during mouse myoblast myogenesis in response to space flight, including expression and secretion. We validated some miRNA expression by qRT-PCR analysis in the limb muscle of a disuse-induced atrophy mouse model, mimicking the unloading following microgravity. Functional assays using miRNA mimics further supported the regulatory roles of some miRNAs in target gene expression, as well as in myoblast proliferation and differentiation. These results suggest that deregulation of these miRNAs may play a functional role in muscle atrophy, and they could be candidate biomarkers of muscle atrophy-associated muscular disease. Importantly, our analysis also suggests that spaceflight may regulate miRNA secretion and retention by modulating RNA-binding proteins (RBPs). Secreted miRNAs originating from skeletal muscle cells have been implicated in various diseases affecting the nervous and skeletal systems. Collectively, these findings highlight the potential role of skeletal muscle-derived miRNAs in systemic adaptation to spaceflight.

## Introduction

Spaceflight has been shown to lead to muscle atrophy, a condition where muscles decrease in size and strength^1^. This phenomenon is similar to what happens on Earth with prolonged bed rest, inactivity, and sedentary human populations such as the elderly^2–4^. Altered expression of gene transcripts matched key biological processes (contractile machinery, calcium homeostasis, muscle development, cell metabolism, inflammatory and oxidative stress response) was mainly reported in the mouse and rat muscle tissue following several days of space flights^5,6^. Therefore, the identification of key regulatory molecules within skeletal muscle cells is not only essential for elucidating the underlying mechanisms of muscle dysfunction but also holds significant potential in providing novel therapeutic targets and preventive strategies for muscle atrophy both in spaceflight and on Earth, including diseases such as sarcopenia and muscular dystrophy.

MicroRNAs (miRNAs) have been implicated as regulators by interacting directly with proteins, or as predictors by secretion for several diseases, such as muscular diseases^7,8^. miRNAs have also been found to be involved in biological responses to microgravity^9,10^. Therefore, identifying miRNAs (and their networks) in response to spaceflight could potentially uncover targets for developing biomarkers and countermeasures to mitigate the associated health risks.

Recent studies based on transcriptomic data from skeletal muscle tissue and circulation in murine models have reported alterations in specific genes or miRNAs caused by spaceflight^10,11^. These were also observed in laboratory animal models of disuse, such as rat and mouse hind limb unloading or tail-suspension models^9^, as well as in humans undergoing bed rest as an analog for spaceflight^12^. However, a detailed molecular characterization of the regulation network of miRNA in skeletal muscle cells due to spaceflight is still lacking. Whether targeting miRNAs can be used as a preventative measure against spaceflight-induced skeletal muscle loss remains to be elucidated.

Here, we cultured mouse skeletal muscle cells C2C12 on board the China Space Station (CSS) and collected the cells and culture medium in orbit. In this study, we provided a systems-level analysis to assess the effects of spaceflight in myocytes by evaluating the transcriptomes and miRNA changes in cells and medium. Our analysis suggested that the alteration of miRNA expression and secretion during spaceflight is associated with the dysfunction of skeletal muscle cells. The regulation of miRNA secretion and retention during spaceflight could be related to the altered expression of RBPs. Some miRNAs may serve as indicators of skeletal muscle physiological status, and might also function as secreted signaling molecules potentially influencing the activities of other tissue cells under spaceflight.

## Results

### Set-up and launch of skeletal muscle cell payload

**Fig. 1:**
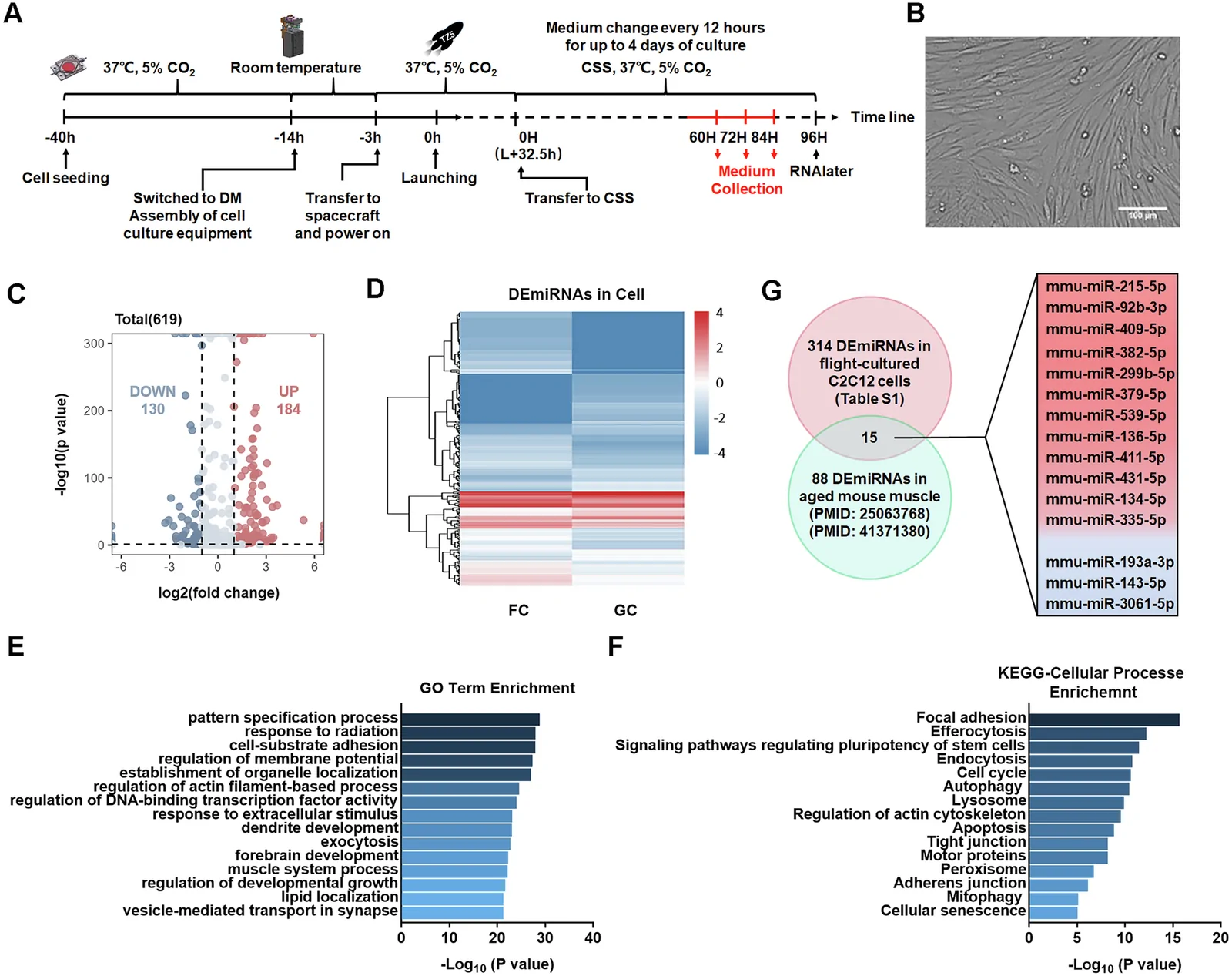
Expression profile of miRNAs in C2C12 during spaceflight. **A** Experimental overview illustrating the procedures for cell loading, hardware installation, cell culture, and sample collection. **B** Representative bright-field image of C2C12 cultured on China Space Station for 24 h. Scale bar, 100 μm. **C** Volcano plots of miRNA-Seq results comparing C2C12 cells under space flight culture (FC) versus ground culture (GC). Red and blue circles represent up- and down-regulated differentially expressed miRNAs (DEmiRNAs; *p* < 0.05, log₂ fold change (log_2_FC) ≥ 1.0), respectively; gray circles indicate non-DEmiRNAs. **D** Heatmap displays the differentially expressed miRNAs in the FC and GC by hierarchical clustering. The color ranges from blue to red indicated the amount of expression (log_10_(norm value)) from low to high. **E** Top 15 GO enrichment terms associated with predicted target genes of DEmiRNAs in FC versus GC. **F** Top 15 KEGG pathway-related cellular processes associated with predicted target genes of DEmiRNAs in FC versus GC. **G** Venn diagram of DEmiRNAs in flight-cultured C2C12 overlayed with those in muscle from aged mice identified previously. 15 miRNAs were found to be either co-down-regulated (blue) or co-up-regulated (red). Fisher’s exact test was applied throughout for assessing differential miRNA expression and for GO and KEGG functional enrichment analyses.

C2C12 mouse skeletal muscle cells were seeded into an autonomous cell culture device, which was engineered by the Shanghai Institute of Technical Physics of the Chinese Academy of Sciences (SITP) and launched aboard the Tianzhou-5 (TZ-5) cargo spacecraft on November 12, 2022, to the China Space Station (CSS) for a 4-day experiment in microgravity. The experimental timeline and task design are illustrated in Fig. 1A. Briefly, C2C12 myoblasts were seeded onto a cell culture chamber 40 h before launch (L-40h). The medium was replaced by differentiation medium (DM), and the cell chambers were connected to a fluid supply bag via infusion tubes equipped with pumps, forming a cell culture module (CCM), 14 h before launch (L-14h) (Fig. 1A). Subsequently, the assembled CCMs were mounted inside a bioreactor that provided electricity, maintained temperature, regulated medium exchange, and controlled the CO₂ concentration at 5%. The bioreactor was transferred to the Tianzhou-5 (TZ-5) cargo spacecraft and powered up 3 h before launch. Following the docking of the TZ-5 cargo spacecraft with the CSS, the CCMs were transferred and installed into a biotechnology experiment cabinet (BTEC) by the crew. Once installed, in-orbit experiments were conducted as described in the “Materials and methods” section.

The first cell image was obtained at 24 h in BTEC culture to show overall cell morphology, confluency, and normal viability (Fig. 1B). The cultured medium was collected at the setting time. After 4 days on CSS, cells were fixed in RNALater in CCM, transferred with the chamber to a 4 °C refrigerating chamber for 12 h, and then stored in a −80 °C freezer with the help of astronauts. The collected cultured medium was transferred directly to the −80 °C freezer. The fixed cells and medium samples from flight culture (FC) aboard the Shenzhou-15 (SZ-15) manned spaceship returned to Earth on June 4, 2023. The ground control (GC) sample used the same protocols in-orbit experiments as described in Materials and Methods. After sample collection, RNA was extracted and purified from each culture for mRNA or miRNA sequence analyses.

### Expression profile of miRNAs in C2C12 during spaceflight

To identify miRNAs in response to spaceflight, we first performed miRNAseq and analyzed the miRNA profiles of FC and GC samples. There are 314 differentially expressed miRNAs (DEmiRNAs) in flight culture compared to the ground control group (Data S1). The space flight led to 184 miRNAs up-regulated and 130 miRNAs down-regulated in C2C12 (Fig. 1C). A heatmap for the identified up- and down-regulated miRNAs is shown in Fig. 1D. To clearly understand the biological functions of these identified DEmiRNAs, potential targets of DEmiRNAs were predicted by TargetScan and miRanda. Then, the overlapping targets were subjected to further Gene Ontology (GO) and Kyoto Encyclopedia of Genes and Genomes (KEGG) pathway enrichment analysis. The GO analysis indicated that the targets of these miRNAs are potentially involved in biological processes described by terms such as “response to radiation”, “regulation of actin filament-based process”, and “muscle system process” (Fig. 1E). KEGG pathway enrichment analysis revealed that the cellular processes identified for the targets of DEmiRNAs are commonly involved in “Regulation of actin cytoskeleton”, “Cell cycle”, “Autophagy”, and “Cellular senescence” (Fig. 1F). These analysis results are consistent with previous reports that space flight can affect cell growth, differentiation, senescence, and autophagy^13–16^, and further indicate that miRNA may serve as a key factor involved in the regulation of these biological processes during space flight.

Spaceflight-induced skeletal muscle dysfunction closely mimics age-related skeletal muscle decline on Earth, with shared alterations in specific molecules and signaling pathways^17,18^. To investigate the potential epigenetic parallels, we retrieved two published skeletal muscle transcriptome datasets from aging mice^19,20^. We then took the union of the dysregulated miRNAs reported in these two studies to generate a combined aging‑associated miRNA signature. Overlapping this signature with our own DEmiRNAs derived from spaceflight-exposed cells, we identified 15 miRNAs that were commonly altered between our dataset and the aging context. Among these, 12 miRNAs were consistently upregulated and 3 miRNAs consistently downregulated in aged muscle relative to young controls (Fig. 1G). Notably, several of these miRNAs (e.g., miR-379-5p, miR-382-5p, miR-134-5p, miR-409-5p) were previously implicated in both muscle atrophy and skeletal muscle dysfunction^21–23^, further supporting the notion that spaceflight may accelerate or mimic certain aspects of muscle aging at the epigenetic level.

### Integrated analysis of differentially expressed miRNA-target modules

**Fig. 2:**
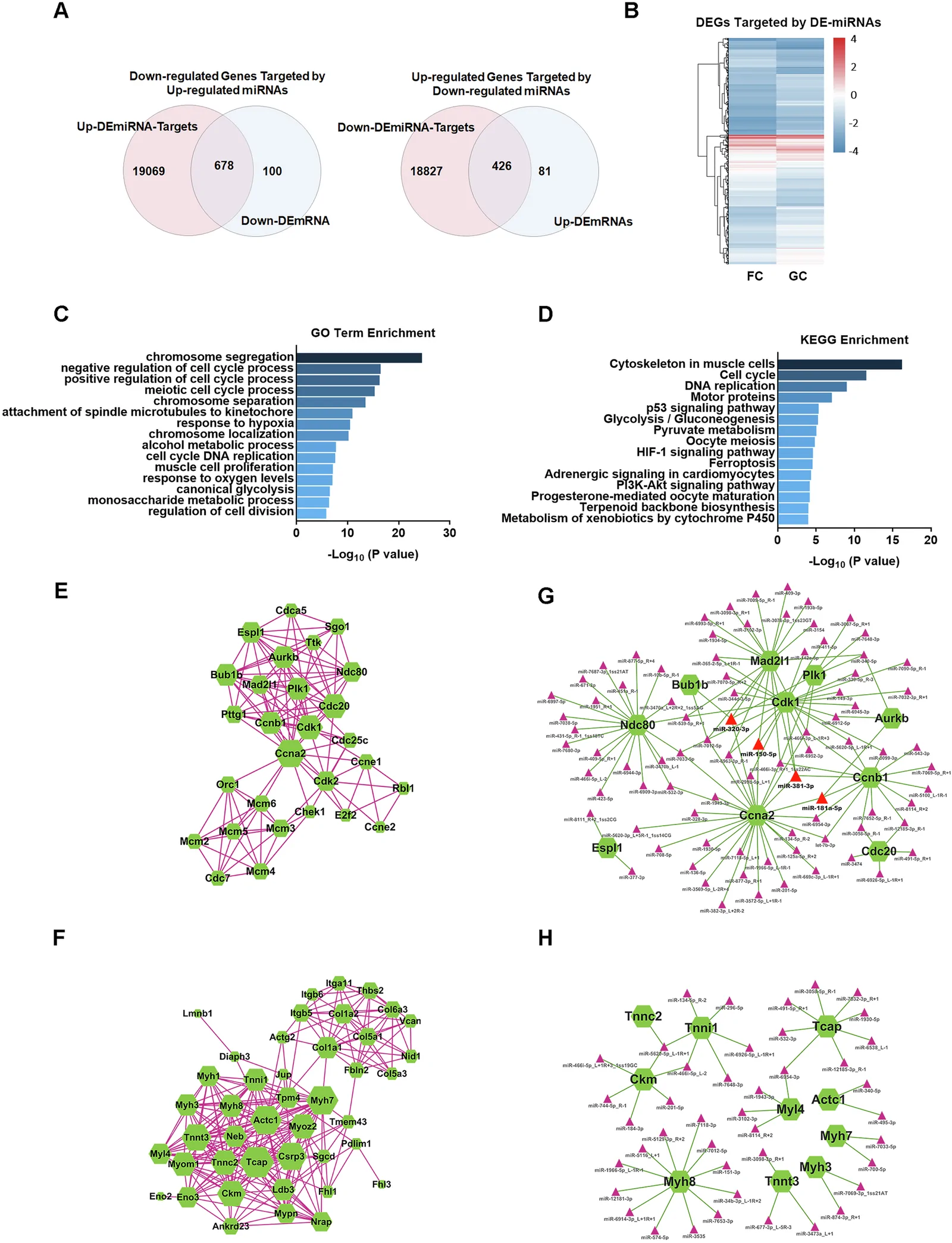
Integrated analysis of differentially expressed miRNA-target modules. **A** Venn diagram showing the overlap between predicted target genes of DEmiRNAs (analyzed using miRanda and TargetScan) and DEmRNAs identified by smart RNA-seq. **B** Heatmap displays the overlapping differentially expressed genes (DEGs) shown in (A) by hierarchical clustering, which are potentially targeted by DEmiRNAs. The color ranges from blue to red indicated the amount of expression (log_10_(norm value)) from low to high. **C** Top 15 GO enrichment terms associated with DEGs targeted by DEmiRNAs in FC versus GC. **D** Top 15 KEGG pathways associated with DEGs targeted by DEmiRNAs in FC versus GC. **E** Protein-protein interaction (PPI) network of cytoskeleton-related genes in muscle cells, identified through KEGG pathway analysis in (**D**). **F.** PPI network of cell cycle-related gene sets, identified via KEGG pathway analysis in (**D**). **G**, **H** miRNA-gene interaction networks illustrating associations between the top 10 hub genes from (**E**, **F**) and their corresponding miRNAs. Red nodes represent miRNAs that are upregulated in both muscular diseases and space flight, purple nodes represent miRNAs that are upregulated in space flight, and green nodes represent the target genes. Fisher’s exact test was applied throughout for assessing differential miRNA expression and for GO and KEGG functional enrichment analyses.

To investigate the transcript abundance of the DEmiRNAs relevant target genes in myocytes, the mRNA expression profiles were confirmed based on Smart-seq of C2C12 cells from the same batch of spaceflight. Considering that miRNAs typically downregulate their target genes, we observed that 678 downregulated genes may be targeted and regulated by the corresponding upregulated miRNAs, while 426 upregulated genes were potentially targeted and regulated by the corresponding downregulated miRNAs (Fig. 2A). A heatmap for the potential negative regulatory genes targeted by DEmiRNAs, identified by Smart-seq, is shown in Fig. 2B (Data S2). GO term and KEGG pathway analysis revealed that the most enriched DEmiRNAs targeted genes are associated with the cell cycle and cytoskeleton in muscle cells (Fig. 2C, D). It suggests that the cell cycle and cytoskeleton can be tightly regulated by miRNAs during spaceflight.

To further identify the hub genes regulated by miRNAs implicated in myogenic function, the gene sets of cell cycle and cytoskeleton in muscle cells (identified via KEGG pathway analysis) were used to construct protein–protein interactions (PPI) network(s) respectively through the STRING interactome database (Fig. 2E, F). The Matthews Correlation Coefficient (MCC) values for each gene were calculated independently, and the top 10 genes were examined for miRNA regulation (Fig. 2G, H). Interestingly, all of these cell cycle and cytoskeleton hub genes were downregulated in the space culture group (Data S2), which may suggest an inhibitory effect of spaceflight on myoblasts expansion and differentiation. The mRNA-miRNA interactions showed that the key regulators are targeted by multiple miRNAs. Furthermore, several miRNAs (such as miR-150-5p, miR-181a-5p, miR-320-3p, and miR-381-3p) previously reported to be upregulated in various muscular disorders diseases^24^, were found to simultaneously target several hub genes (Fig. 2G, H). This observation raises the possibility that spaceflight-associated changes in C2C12 may share certain features with terrestrial muscle disorders, potentially involving in the upregulation of specific miRNAs. These miRNAs can be further explored as candidate therapeutic targets for muscle atrophy both in spaceflight and on Earth.

### Spaceflight regulates miRNA enrichment in the medium

**Fig. 3:**
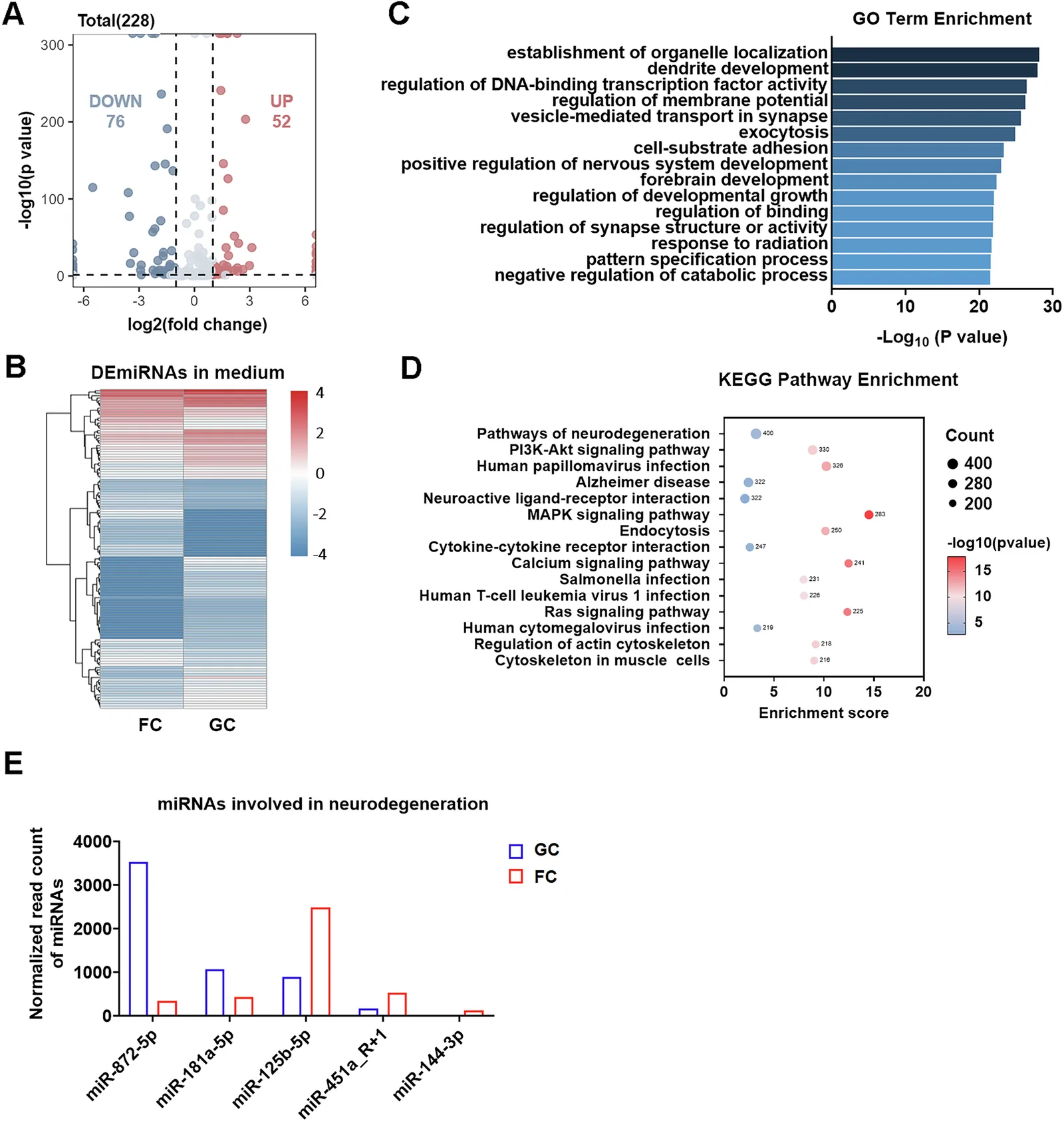
Spaceflight regulates miRNA enrichment in the medium. **A** Volcano plot of DE-miRNAs from C2C12 culture medium (space flight vs. ground). Red/blue circles show significantly up-/down-regulated miRNAs (*p* < 0.05, log₂FC ≥ 1.0); gray circles show non-significant miRNAs. **B** Heatmap displays DE-miRNAs from C2C12 culture medium (space flight vs. ground) by hierarchical clustering. The color ranges from blue to red indicated the amount of expression (log_10_(norm value)) from low to high. **C** Top 15 GO terms for target genes of DE-miRNAs from culture medium. **D** Top 15 KEGG pathways for target genes of DE-miRNAs from culture medium. **E** Neurodegeneration-associated miRNAs dysregulated by space flight in culture medium, corresponding to (**B**). Fisher’s exact test was applied throughout for assessing differential miRNA expression and for GO and KEGG functional enrichment analyses.

In addition to the effects on cellular miRNAs, spaceflight has a dramatic impact on circulating miRNA abundance^25^. Thus, the medium after culture in orbit was collected at the designated time points (Fig. 1A), and the miRNA sequencing was performed. The 228 miRNAs can be detected in the culture medium, which were secreted by C2C12 cells. 52 and 76 miRNAs were upregulated and downregulated after space culture, respectively (Fig. 3A and Data S3). A heatmap for the identified DEmiRNAs derived from medium is shown in Fig. 3B. To explore the possibility that miRNAs secreted during spaceflight modulate biological processes or pathways, we performed GO term and KEGG analyses using predicted target genes by medium miRNAs (Fig. 3C, D). Of note, we observed that the genes targeted by DEmiRNAs in the culture medium are involved in the regulation of the neuronal system and neurodegenerative diseases, in addition to affecting the skeletal muscle system.

Among the DEmiRNAs from the medium, miR-872 and miR-181a, which are both positive regulators of neural stem cells and enhance the learning and memory of aged mice^26,27^, were down-regulated after spaceflight. miR-125b, miR-144 and miR-451, which are known as important factor of Alzheimer’s disease progression^28,29^, were all upregulated in space flight medium (Fig. 3E). In fact, spaceflight can have numerous effects on the central nervous system, including brain structure and function^30,31^. Together, these analyses support the notion that miRNAs secreted from skeletal muscle during spaceflight could function as signaling molecules, potentially contributing to muscle atrophy and influencing the physiology of other systems, such as the nervous system.

### MiRNA secretion of C2C12 in response to spaceflight

**Fig. 4:**
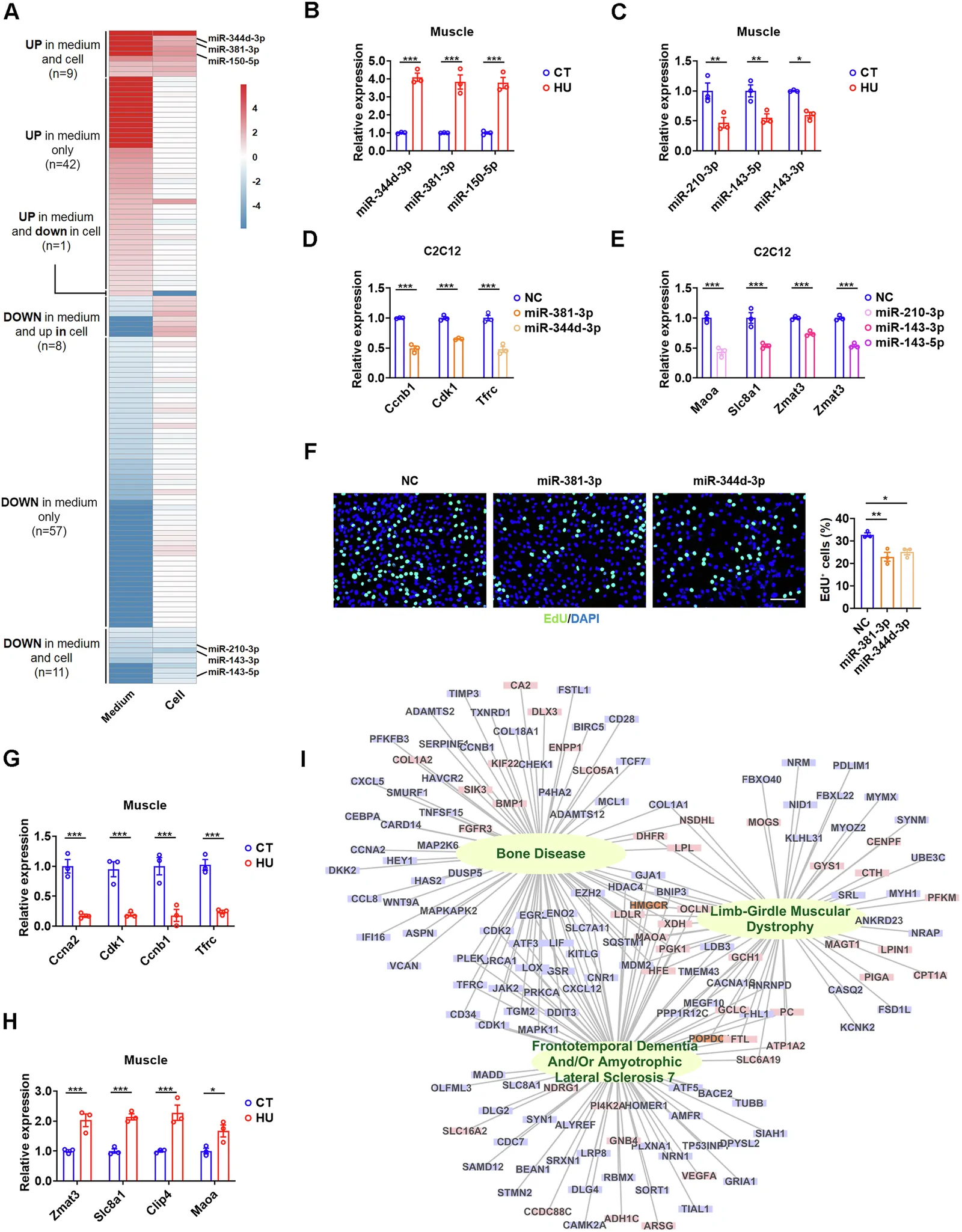
MiRNA secretion of C2C12 in response to spaceflight. **A** Heatmap showing the log_2_FC of spaceflight-regulated miRNAs in C2C12 medium (left) and their expression in cells (right). The heatmap is sorted by log_2_FC (descending), and the color scale represents average Z score. Spaceflight-upregulated miRNAs (**B**) and downregulated miRNAs (**C**) detected in muscles of the hindlimb unloading (HU)-induced muscle atrophy model compared with control (CT). **D**, **E** qPCR analysis of miRNA-predicted targets in C2C12 cells transfected with miRNA mimics or negative control (NC). **F** EdU staining on C2C12 cells transfected with miRNA mimics or NC, and quantification of the percentage of EdU^+^ cells. Scale bar, 100 μm. **G**, **H** qPCR analysis of the potential target genes in muscle of the HU-induced muscle atrophy model. **I** Disease analysis of predicted target genes of both up- and down-regulated miRNAs in cells and medium using GeneAnalytics (pink denotes differentially expressed genes in diseases). For (**B**–**H**), data represent mean ± SD. Statistical analysis was performed by t test (*p < 0.05; **p < 0.01; ***p < 0.001).

Actually, the expression of miRNA in the culture medium may simply reflect cellular expression and be actively secreted, or it may be passively regulated by other molecules within the cell for secretion or retention. As noted above, among the 128 miRNAs in medium regulated by space flight, a total of 20 (15.6%) miRNAs were co-regulated in cells, with 9 being co-upregulated and 11 being co-downregulated (Fig. 4A and Data S4), suggesting that the relative abundance of miRNAs in culture medium can reflect the expression of cells. We evaluated the expression level of co-regulated miRNAs in skeletal muscle of the hindlimb unloading (HU)-induced muscle atrophy model, which has been widely used to model the effects of microgravity (Fig. 4B, C). Among co-upregulated miRNAs, both miR-381-3p and miR-150-5p were reported to inhibit myoblast proliferation, and circulating miR-150-5p can act as a potential biomarker for myasthenia gravis^32–34^. Low levels of miR-143-3p and miR-210-3p are associated with myoblast senescence or sarcopenia^35,36^. Furthermore, enrichment of miR-210-3p and miR-143-5p promoted skeletal muscle regeneration and supported the anti-atrophic effects in mice, respectively^37,38^.

To further confirm whether the miRNAs found to be dysregulated in spaceflight exert regulatory effects on their predicted targets (Fig. S1A, B), miRNA mimic transfection experiments were performed (Fig. S1C). Notably, bioinformatic analyses predicted that miR-381-3p may simultaneously target multiple key cell cycle–related genes (Ccna2, Ccnb1, Cdk1) (Fig. S1A); but mimics transfection only significantly downregulated Ccnb1 and Cdk1 (Fig. 4D). Ccna2 was predicted as a potential target of miR-150-5p (Fig. S1B), yet not downregulated by its mimics (Fig. S1D). miR-344d-3p downregulated its predicted target Tfrc as expected (Fig. 4D). Zmat3 was predicted as a shared target of miR-143-3p/5p and miR-210-3p, yet only miR-143-3p and miR-143-5p significantly downregulated it (Fig. 4E). The predictions that miR-210-3p targets Maoa and miR-143-3p targets Scl8a1 were both validated by mimics transfection (Fig. 4E).

Subsequently, we further evaluated the effects of these validated miRNAs on myoblast function, including proliferation and differentiation. The results showed that transfection with miR-381-3p significantly decreased the percentage of EdU‑positive myoblasts (Fig. 4F), consistent with its suppression of cell cycle‑related target genes (Fig. 4D). Transfection with miR-344d-3p reduced the mRNA level of its predicted target Tfrc (Fig. 4D) and markedly decreased both the percentage of EdU‑positive myoblasts (Fig. 4F) and the number of myotubes formed (Fig. S1E, F), consistent with a previous report showing that Tfrc deletion in muscle stem cells results in irreversible stem cell depletion and cell‑autonomous defects in proliferation and differentiation, leading to impaired skeletal muscle regeneration^39^. Transfection with miR-210-3p, miR-143-3p or miR-143-5p mimics all resulted in reduced myotube formation (Fig. S1E, F) and lower expression of fusion-related genes compared with control cells (Fig. S1G), suggesting an inhibitory effect on differentiation. Notably, previous study has shown that miR-143-3p inhibits myogenic differentiation^40^ consistent with our results. Given that Zmat3, a p53-induced gene, contributes to cellular senescence and the activation of the p53/p21 pathway^41,42^, and that Maoa is associated with skeletal muscle atrophy through the generation of reactive oxygen species and the activation of pro-inflammatory signaling pathways^43^, it is suggested that although these three miRNAs inhibit cell differentiation, they may also suppress cellular senescence by targeting genes. Nevertheless, this possibility needs to be further confirmed by future studies.

In addition, these predicted target genes were found to be regulated in the skeletal muscle of HU mice in a manner consistent with their changes observed during spaceflight (Fig. 4G, H). Collectively, these findings suggest that miRNAs may contribute to disuse muscle atrophy by regulating processes such as cell cycle progression and cellular senescence, thereby supporting their potential as both biomarkers and therapeutic targets for countering unloading-induced muscle wasting.

Likewise, to understand the role of potential target genes of co-upregulated and co-downregulated miRNAs in cell and medium relevant with disease pathology, we used GeneAnalytics^44^ to identify these target genes associated diseases. As shown in Fig. 4I, these potential target genes were predicted to be related to Limb-Girdle Muscular Dystrophy, Bone disease, and Frontotemporal Dementia and/or Lateral Sclerosis.

### Spaceflight regulates miRNA secretion and retention by modulating RBP

However, 108 (84.3%) miRNAs from the medium were only modulated by spaceflight and were not similarly regulated in cells (Fig. 4A). Among these miRNAs, 9 miRNAs were significantly regulated in the opposite direction in medium and cells, with only one miRNA being upregulated in medium but downregulated in cells. 8 miRNAs were downregulated in medium but upregulated in cells (Fig. 4A), suggesting that the secretion of these miRNAs might be inhibited and retained in the cells after spaceflight. A total of 99 miRNAs were changed only in the medium, including 42 upregulated and 57 downregulated (Fig. 4A). Of these, 48 were spaceflight-regulated miRNAs that showed no change in cells, while the remaining 51 that were merely modulated in the medium and were not detectable in the cells (Fig. 4A and Data S4), demonstrating that in these cases, the secretion activities of these miRNAs had been affected after spaceflight, and many of them may be mainly produced for secretion and action on other potential distal target cells.

**Fig. 5:**
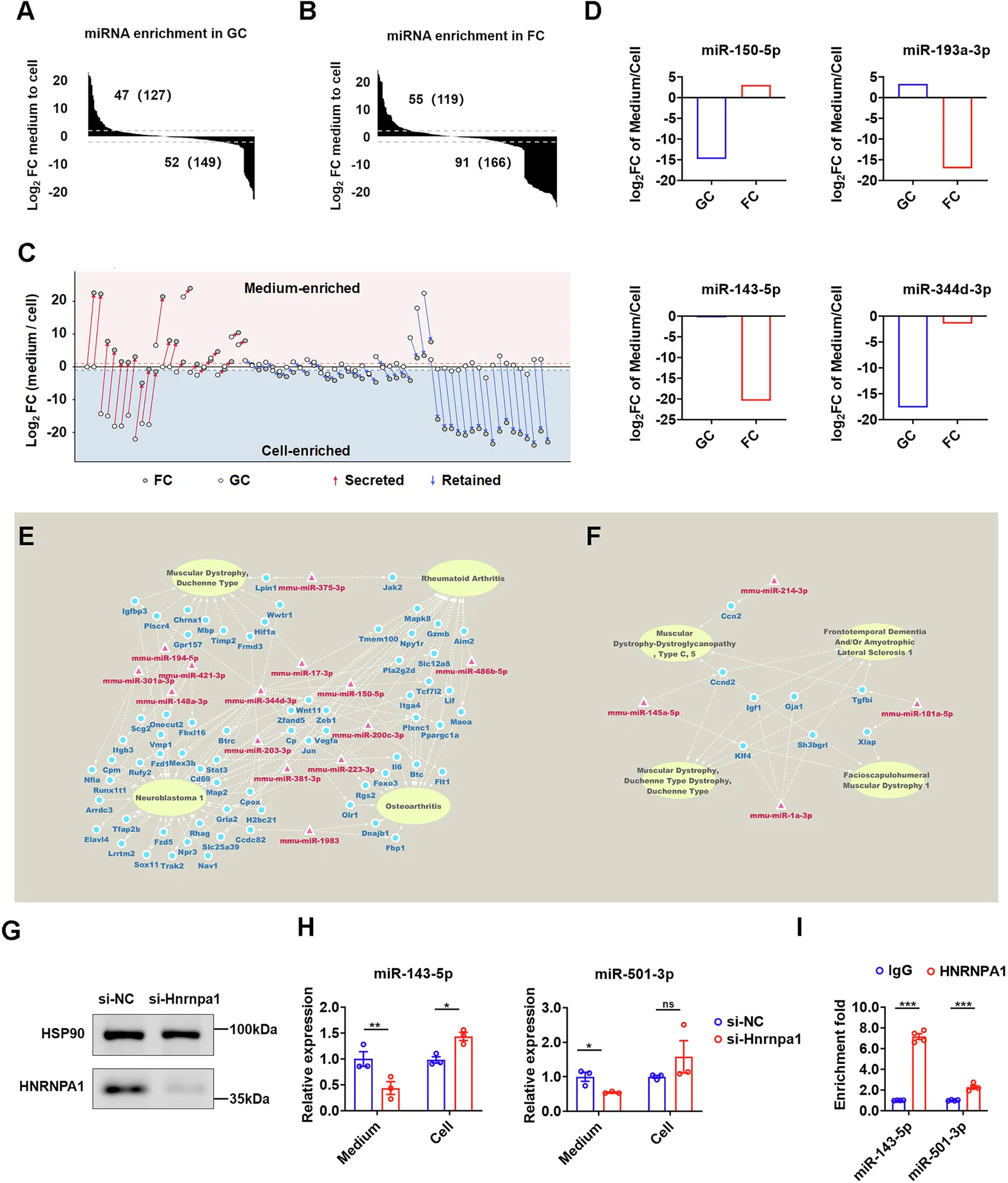
Spaceflight regulates miRNA secretion and retention. **A** Waterfall plot of medium-enriched (log_2_FC ≥ 2, *n* = 47) and cell-enriched (log_2_FC ≤ −2, *n* = 52) miRNAs under ground culture conditions. **B** Waterfall plot of medium-enriched (log_2_FC ≥ 2, *n* = 55) and cell-enriched miRNAs (log_2_FC ≤ −2, *n* = 91) under spaceflight culture conditions. **C** Changes in miRNA enrichment induced by spaceflight, where cell-enriched miRNAs are defined by log_2_FC(medium/cell) ≤ −2 and medium-enriched miRNAs are defined by log_2_FC(medium/cell) ≥ 2. Arrows connect the same miRNAs under ground control (GC, white dots) and spaceflight conditions (FC, gray dots), with red indicating miRNAs secreted upon spaceflight and blue indicating those retained. **D** Bar plots showing miRNA enrichment levels under ground and spaceflight conditions for representative miRNAs. Disease analysis of predicted target genes of secreted miRNAs (**E**) and retained miRNAs (**F**) using GeneAnalytics. (Red indicates miRNAs differentially secreted or retained under spaceflight; blue highlights experimentally validated target genes from published literature; yellow denotes target genes associated with disease.) **G** Proteins were measured by western blot in C2C12 cells transfected with negative control siRNA (si-NC) or si-Hnrnpa1. **H** Cellular and medium expression of miR-143-5p and miR-501-3p in C2C12 cells transfected with si-NC or si-Hnrnpa1. **I** RNA immunoprecipitation (RIP) was performed in C2C12 cells using an antibody against HNRNPA1, and the retrieved miRNAs were analyzed by qRT-PCR. Enrichment was calculated as the level of miRNAs associated with HNRNPA1 relative to that of the IgG control. For (**H**, **I**), data represent mean ± SD. Statistical analysis was performed by t test (*p < 0.05; **p < 0.01; ***p < 0.001; ns not significant).

Comparison of the relative levels for each miRNA in the cell versus medium revealed unique patterns of miRNA secretion and retention^45^. We noticed that in the ground culture group, 47 miRNAs were significantly enriched in medium defined as 4-fold levels in medium versus cells (log_2_FC of medium/cell ≥ 2), while 52 miRNAs were enriched in cells with 4-fold levels in cells versus medium (log_2_FC of medium/cell ≤ −2) (Fig. 5A). After spaceflight, 55 miRNAs were medium-enriched, and 91 miRNAs were cell-enriched (Fig. 5B). To better understand the effect of spaceflight on miRNA enrichment, we calculated a medium/cell-enrichment score of each miRNA in the spaceflight versus the ground state (Fig. 5C and Data S5). This revealed 23 miRNAs in medium regulated by spaceflight that showed significantly higher medium-enrichment scores following spaceflight, while 44 miRNAs in medium regulated by spaceflight showed greater cellular retention in response to spaceflight (Data S5). For example, miR-150-5p showed 14.8-fold cell enrichment in the ground but became 3.1-fold medium enriched following spaceflight culture (Fig. 5D). Conversely, miR-193a-3p showed 3.3-fold medium enrichment in the ground but became 17-fold cell enriched following spaceflight culture (Fig. 5D). Likewise, spaceflight caused miR-143-5p to become cell enriched, whereas the basally cell-enriched miR-344d-3p lost its enrichment after spaceflight (Fig. 5D).

To explore the pathological relevance of secreted and retained miRNAs, we first retrieved their luciferase- and CLIP-validated target genes with the multiMiR package. These high-confidence targets were then subjected to disease-enrichment analysis using the GeneAnalytics suite (Data S6 and Data S7). The results indicated that experimentally validated target genes of secreted miRNAs were significantly enriched in diseases such as Muscular Dystrophy (Duchenne Type), Rheumatoid Arthritis, Osteoarthritis, and Neuroblastoma (Fig. 5E). Whereas, target genes of retained miRNAs showed stronger associations with skeletal muscle atrophy disorders (Fig. 5F). These analyses suggested that secreted miRNAs play a role in intercellular communication, potentially modulating disease-related pathways in distant tissues, as their target genes have been found to be associated with muscular, inflammatory, and neurodegenerative disorders. In contrast, retained miRNAs appear to be more involved in regulating localized processes such as skeletal muscle atrophy.

Previously, people have shown that RBPs recognize specific sequences within miRNAs, thus favoring their sorting into extracellular vesicles (EVs) or their retention in the cell^45,46^. To determine which RBPs are involved in regulating miRNA sorting in spaceflight, we first performed the binding prediction of differentially expressed RBPs on secreted and retained RNA sequences (Table 1). The downregulated Hnrnp family members, such as Hnrnpa1 and Hnrnpa2b1, were predicted to bind to most retained miRNAs. To further explore the potential role of RBPs in regulating miRNA secretion or retention, we performed siRNA-mediated knockdown of Hnrnpa1 and Hnrnpa2b1 (Figs. 5G and S2A) and examined miRNA expression in cells and culture medium. Knockdown of Hnrnpa1 and Hnrnpa2b1 both significantly reduced the levels of miR-501-3p in the culture medium, while its intracellular levels remained unchanged (Figs. 5H and S2B). In addition, knockdown of Hnrnpa1 also reduced the levels of miR-143-5p in the culture medium but increased its level in cells (Fig. 5H). To further investigate possible physical interactions, a native RNA immunoprecipitation (RIP) assay was performed using antibody against HNRNPA1 (Fig. S2C). Higher levels of miR-143-5p and miR-501-3p were pulled down by the HNRNPA1 antibody compared with the IgG control (Fig. 5I). Taken together, these data suggest a possible role for Hnrnpa1 and Hnrnpa2b1 in modulating miRNA release or retention and dysregulated RBPs during spaceflight may participate in the differential distribution of miRNAs between cells and culture medium.

**Table 1:** Spaceflight regulates miRNA secretion and retention by modulating RBP

RBP name	Binding secreted miRNAs	Binding retained miRNAs	Fold change	*P* value
Pcbp1	miR-150-5p miR-744-3p_R-1	miR-351-5p_R-3 miR-296-5p miR-193a-3p miR-574-3p miR-3110-5p	2.34	3.54E-09
Fmr1	miR-381-3p		1.83	2.23E-05
Rbm42	miR-344d-3p miR-744-3p_R-1 miR-148a-3p	miR-1a-3p miR-151-3p miR-3535	1.70	2.00E-04
Ilf2		miR-872-5p miR-296-3p miR-501-3p_R + 1 miR-744-5p_R-1 miR-664-5p_R + 1	0.66	4.46E-03
Hnrnpm	miR-200a-3p_R + 1 miR-223-3p miR-375-3p	miR-872-5p miR-296-3p miR-181a-5p	0.60	3.63E-04
Hnrnpa2b1	miR-301a-3p miR-17-3p	miR-872-5p miR-296-3p miR-501-3p_R + 1 miR-744-5p_R-1 miR-126a-3p_R-1 miR-664-5p_R + 1 miR-1839-5p	0.59	2.56E-04
Srsf7	miR-203-3p	miR-3535	0.53	1.46E-05
Srsf1		miR-296-3p miR-378a-3p_R + 1 miR-1843a-5p_L-1R + 1	0.51	3.58E-06
Hnrnph1		miR-296-3p miR-351-3p	0.45	3.00E-08
Hnrnpa1	miR-203-3p miR-223-3p miR-301a-3p miR-17-3p	miR-872-5p miR-322-5p miR-501-3p_R + 1 miR-143-5p miR-664-5p_R + 1	0.40	3.49E-10

Binding analyses of RBPs and miRNAs were performed using RBPmap on miRNAs that were either secreted or retained under spaceflight conditions. This analysis included 10 RBPs that were differentially expressed in cells from ground control (GC) versus spaceflight culture (FC). Their fold changes and P-values are presented in the table.

## Discussion

Skeletal muscle undergoes atrophy and adaptive remodeling during spaceflight, which not only compromises its structural and functional integrity but also facilitates cross-organ communication through the secretion of myokines, thereby influencing the adaptive responses of other tissues and organs to microgravity. In this study, we focus on the changes in miRNA expression both inside muscle cells and in the culture media following spaceflight. Findings from this study uncover that the dysregulated miRNAs are predicted to target key genes governing myogenesis, which could potentially contribute to the progression of muscle atrophy.

Plasma miR-210 decreases in response to sarcopenia in the elderly and delivery of miR-210-3p targeting KLF7 stimulates MuSCs activation and promotes skeletal muscle regeneration in sarcopenia^36,37^. In C2C12 cells subjected to spaceflight, miR-210-3p and Klf7 were both downregulated. However, bioinformatic analysis and miRNA overexpression analysis identified Maoa could be a potential target of miR-210-3p during spaceflight (Figs. 4E and S1B). Notably, miR-143-5p could target Zmat3, acts as an effector of p53, which was significantly upregulated during space flight^10^. ZMAT3 can promote TNF-α mRNA processing and activate the NF-κB signaling pathway, impare the self-renewal ability of hematopoietic stem cells (HSCs) and reduce the HSCs differentiation into myeloid hematopoietic cells^47^, and the canonical NF-ĸB signaling pathway negatively regulates myogenesis^48^. Based on the consistent expression results in C2C12 (Fig. 4E) and HU model (Figs. 4C and 4H), we speculate that the low level of miR-143-5p can maintain a high level of Zmat3, which could play a role in muscle atrophy induced by unloading in tail suspension and spaceflight condition.

Low levels of Tfrc have been reported to be closely related to impaired skeletal muscle development and regeneration, as well as denervation-induced muscle atrophy and skeletal muscle aging^39,49^. Tfrc is predicted to be a target of miR-344d-3p (Figs. 4D and S1A). Therefore, modulating miR-344d-3p to regulate Tfrc represents a potential therapeutic strategy for improving skeletal muscle function in the context of atrophy or aging.

Previous studies have suggested that during aging, the downregulation of miR-143-3p mediated through targeting Igfbp5 may represent a compensatory mechanism aimed at enhancing myogenic efficiency^35^. In C2C12 myoblasts subjected to spaceflight conditions, miR-143-3p was indeed downregulated, while our analysis identified Slc8a1 and Zmat3 as other potential targets of miR-143-3p (Figs. 4E and S1B). In addition, we observed that miR-143-3p overexpression inhibited myoblast differentiation. Thus, we cannot rule out the possibility that downregulation of miR-143-3p in spaceflight may reflect additional functions or serve as a compensatory response to cellular dysfunction.

This cell-autonomous regulatory network likely constitutes a fundamental mechanism underpinning the functional decline of skeletal muscle itself. More importantly, our bioinformatic analyses reveal that many of the differentially secreted miRNAs have predicted targets involved in neuronal and bone health. The distinct expression of miRNAs identified in the medium during flight strongly suggests their active secretion, likely via EVs or other ribonucleoprotein complexes. This observation implies that skeletal muscle not only undergoes intrinsic remodeling but also dispatches specific molecular signals to the extracellular milieu. This finding suggests a potential role of muscle-derived miRNAs as mediators of inter-tissue crosstalk, which may contribute to the multifaceted physiological changes associated with spaceflight. We tentatively propose that the selective packaging and release of these miRNAs could represent a mechanistic link between muscle disuse and the pathophysiological changes observed in other organ systems, although further validation is needed.

We acknowledge several limitations of this study. First, owing to the inherent constraints of space research, only one flight mission was conducted, and the overall sample number was limited. Second, many of our findings were derived solely from data analyses and relevant literature reports, without direct experimental validation. Although we performed preliminary validation for representative miRNAs and their targets, direct miRNA–target interactions require further verification (e.g., luciferase reporter assays and site-directed mutagenesis). Moreover, broader functional analyses remain to be addressed; for example, senescence‑related assessments (such as SA‑β‑gal staining, γH2AX staining, or p16 expression analysis) are needed to fully understand the biological consequences of dysregulated miRNAs. Third, uncontrolled confounding factors inherent to the spaceflight environment, such as altered fluid exchange efficiency under microgravity, may have influenced the results and should be taken into account when interpreting the data. Additionally, functional validation using co‑culture models or in vivo delivery of specific miRNAs is required to confirm the physiological relevance of the proposed miRNA‑mediated communication axis. Future studies with independent flight opportunities and more comprehensive controls are needed to extend our observations.

Despite these limitations, our data delineated a complex, miRNA-mediated communication axis originating from skeletal muscle cells in response to spaceflight. Our work firmly established the significance of the muscle’s secretory repertoire. Understanding this regulatory circuitry not only deepens our fundamental knowledge of systemic physiology in extreme environments but also unveils potential circulating miRNA biomarkers or therapeutic targets for mitigating the multisystem deconditioning experienced by astronauts.

## Methods

### Hardware specifications

The cell culture equipment was designed and manufactured by the Shanghai Institute of Technical Physics of the Chinese Academy of Sciences. The equipment consisted of culture chambers, liquid storage bags, cell culture modules, and an electronic container. Each culture chamber (28 × 26 × 11 mm) was loaded with 3 mL of medium. Two medium storage bags were filled with differentiation medium and RNALater (Byotime Biotechnology). Two empty bags were used for culture medium collection and medium waste recovery. As a fully automated system, cell culture modules provided temperature control, microscope imaging, medium recirculation, and medium routing via micro peristaltic pumps and pinch valves. The cells were cultured at 37 ± 0.5 °C, and the storage bags were stored at 4 ± 0.5 °C. The device automatically changed liquids at predetermined time points at a rate of 0.6 ml/min.

### Cell culture

Mouse C2C12 myoblasts were obtained from American Type Culture Collection (ATCC) and cultured in growth medium, consisting of DMEM medium (Gibco) with 10% fetal bovine serum (Gibco), 1% penicillin/streptomycin (Gibco) in a 5% CO_2_ humidified incubator at 37 °C. When C2C12 myoblasts grew up to 90% confluent, myoblasts were transferred to a differentiation medium (DM) consisting of DMEM with 2% horse serum (16050-114, Gibco) and 1% penicillin/streptomycin.

Cell transfection was performed using Lipofectamine 2000 (Invitrogen) according to the standard protocol. For proliferation analysis, cells were treated with 10 µM EdU (C10086, Thermo Fisher Scientific) for 3 h and EdU incorporation was detected using the Click-iT™ EdU Imaging Kit (Thermo Fisher Scientific) following the manufacturer’s instructions. GMR-miR™ microRNA double-stranded mimics for miR-381 - 3p (UAU ACA AGG GCA AGC UCU CUG U), miR-210 - 3p (CUG UGC GUG UGA CAG CGG CUG A), miR-150 - 5p (UCU CCC AAC CCU UGU ACC AGU G), miR-344 d - 3p (GAU AUA ACC ACU GCC AGA CUG A, miR-143 - 3p (UGA GAU GAA GCA CUG UAG CUC), miR-143 - 5p (GGU GCA GUG CUG CAU CUC UGG), mimics control (UUC UCC GAA CGU GUC ACG UTT), siRNA oligos for hnRNPA1 (CGA UGA AGG GAG GAA ACU UTT) and siRNA oligos for hnRNPA2B1 (GGU GGA AUU AAG GAA GAU ATT) were obtained from GenePharma.

### Immunofluorescence

Cells were fixed in 4% PFA for 15 min and permeabilized with 0.5% NP-40 for 10 min. Then cells were blocked in 5% BSA for 1 h followed by incubating with primary antibodies overnight at 4 °C and secondary antibodies for 1 h at room temperature (RT). Antibodies and dilutions were used as follows: mouse anti-MyHC (1:100, Developmental Studies Hybridoma Bank); goat anti-mouse IgG Alexa Fluor 488 (1:200, Invitrogen). Images were captured by a fluorescence microscope (Cycloud Bio).

### Space experiment

Two days before TZ-5 launch, C2C12 myoblasts were seeded in collagen-coated culture chambers. Then the growth medium was switched to a differentiation medium, and culture chambers were assembled into the cell-culture equipment 14 h before launch. On the first day after launch, the culture medium was replaced with fresh culture medium. When the CCM was transferred and installed into a biotechnology experiment cabinet (BTEC), the culture medium was replaced with fresh culture medium. During 4 days of spaceflight, the medium was changed every 12 h. Collect the culture medium when changing the medium for the last three times. The ground cells were cultured in conventional incubators, but were handled otherwise in the same manner and with the same time stamps as in space culture.

### Mouse studies

All animal experiments were performed in accordance with guidelines of the Ethics Committee of Shanghai Institute of Nutrition and Health (SINH), Chinese Academy of Sciences (CAS), and approved by the Animal Care Committee of SINH (SINH-2025-YH-3). All mice were housed at a temperature of 23 ± 3 °C and humidity of 35 ± 5% under a 12 h light/dark cycle in a specific pathogen-free animal facility. Male mice with a C57BL/6J background at 8 weeks of age were randomly divided into the suspension (HU) group or the control (CT) group. In the HU model, the tail one-third of the mouse’s near-body side is wrapped with tape, and the tail is suspended with nylon clips to fix the mouse’s tail on the top of the cage cover, keeping the mouse’s body at an angle of 30–40° to the ground. The front limbs can touch the bottom of the cage, while the hind limbs are suspended in the air. Ensure that the mice can move freely, eat, and drink water with their heads at a low position in the cage. During the tail suspension process, closely monitor the condition of the mouse, regularly check the blood circulation in the mouse’s tail, and promptly adjust the suspension height to maintain the best angle. The CT group was not suspended and could move freely, eat, and drink. After being suspended for 2 weeks, all the mice were euthanized by cervical dislocation, and hind limb muscles were collected.

### RNA isolation and real-time PCR

After cell samples fixed with RNALater were collected, RNALater was removed by centrifugation at 10,000 × *g* for 3 min. Gastrocnemius muscles were dissected from the mouse model immediately after euthanasia, snap-frozen in liquid nitrogen, and stored at −80 °C until processing. For RNA extraction, frozen muscle tissue was homogenized in 1 mL of TRIzol reagent (Invitrogen) using a tissue homogenizer. Total RNA was isolated by using Trizol reagent (Invitrogen) according to the manufacturer’s instructions. The prepared RNA samples were subjected to deep sequencing. Five hundred nanograms of RNA were reverse-transcribed with the miRNA 1st-Strand cDNA Synthesis Kit (Accurate Biology, AG11716) according to the manufacturer’s instructions. miRNA-specific forward primers were designed in-house; the universal reverse primer (complementary to the adapter ligated during reverse transcription) is supplied within the same kit. Real-time PCR was performed on QuantStudio 6 (Applied Biosystems). Data were analyzed using the ΔΔCt method with values normalized to U6 (for miRNA) or GAPDH (for mRNA). Primers used in this study were provided in Table S1.

### Native RNA immunoprecipitation (RIP) assay

Briefly, cells were lysed in 100 μl lysis buffer (100 mM KCl, 5 mM MgCl₂, 10 mM HEPES‑KOH pH 7.0, 0.5% NP‑40, 1 mM DTT, 100 U/ml RNaseOUT, 400 μM VRC, and protease inhibitors). Protein A/G PLUS‑Agarose beads (40 μl per reaction) were washed with NT2 buffer (50 mM Tris‑HCl pH 7.4, 150 mM NaCl, 1 mM MgCl₂, 0.05% NP‑40), incubated with 5 μg antibody for 1 h at RT, washed five times with NT2 buffer, and resuspended in NT2 buffer supplemented with 200 U RNaseOUT, 400 μM VRC, 10 μl of 100 mM DTT, and 20 mM EDTA. Cell lysate (100 μl) was added to the antibody‑coated beads and rotated overnight at 4 °C. The next day, beads were washed five times with NT2 buffer, resuspended in 100 μl NT2, and 10 μl was saved for protein analysis. Total RNA was extracted from the remaining volume using 1 ml TRIzol.

### Western blot analysis

Cells were extracted by using RIPA buffer (Beyotime) supplemented with Protease Inhibitor Cocktail (Roche) and Phosphatase Inhibitor Cocktail (Roche) immediately before use and the protein concentration was determined using a BCA Kit (Beyotime). Rabbit anti-HSP90(4874S, CST) and rabbit anti-HNRNPA1 (A11564, ABclonal) antibodies were used. All blots are provided in Fig. S3.

### Small RNA library construction and deep sequencing

To compare miRNA profiles between space- and ground-cultured cells, four groups of samples were processed in parallel: space-flight cells, space-flight culture medium, ground control cells, and ground control culture medium. Total RNA from each group was subjected to library construction using the Illumina TruSeq Small RNA Preparation Kit (LC Sciences, Hangzhou, China) according to the manufacturer’s instructions. Briefly, RNA was ligated to 3′ and 5′ adapters, reverse-transcribed, and PCR-amplified to generate cDNA constructs. Fragments of 18–30 nt were size-selected on a 6% denaturing polyacrylamide gel, purified, and validated. The final libraries were clustered and sequenced on an Illumina Solexa platform. Raw reads were processed with Illumina analysis software, and downstream analyses were performed using the ACGT101-miR pipeline (version 4.2; LC Sciences). Data normalization followed the procedures as described in a previous study^50^ with minor modifications. The normalized read count of some miRNAs was set to be 0.0001 for further calculation if it has no reads in the library. Using ground-control samples as the reference, differential miRNA expression was assessed by Fisher’s exact test (Poisson model, ACGT101-miR pipeline; LC-Bio), and significance was defined as |log2FC| ≥ 1 and *p* ≤ 0.05.

### Sequence analysis and identification of known and novel miRNAs

The raw reads after removing adapter dimers, junk, low complexity, common RNA families (rRNA, tRNA, snRNA, snoRNA), and repeats. The unique clean reads with length in 18–26 nt were mapped to specific species precursors in miRBase 22.1 by BLAST search to identify known miRNAs. The criteria used for known miRNA annotation and hairpin structure determination as follows: (1) L-n means the miRNA_seq (detected) is n base less than known rep_miRSeq in the left side; (2) R-n means the miRNA_seq (detected) is n base less than known rep_miRSeq in the right side; (3) L + n means the miRNA_seq (detected) is n base more than known rep_miRSeq in the left side; (4) R + n means the miRNA_seq (detected) is n base more than known rep_miRSeq in the right side; (5) 2ss5TC13TA means 2 substitut in (ss), which are T- > C at position 5 and T- > A at position 13; (6) if there is no matching annotation, the miRNA_seq (detected) is exactly same as known rep_miRSeq.

### Smart RNA-seq analysis

SMARTer cDNA synthesis starts with picogram amounts of total RNA. A modified oligo(dT) primer (the SMART CDS Primer) primes the first-strand synthesis reaction. When SMARTScribe^TM^ Reverse Transcriptase reaches the 5′ end of the mRNA, the enzyme’s terminal transferase activity adds a few additional nucleotides to the 3′ end of the cDNA. The carefully-designed SMARTer Oligonucleotide base-pairs with the non-template nucleotide stretch, creating an extended template to enable SMARTScribe RT continue replicating to the end of the oligonucleotide. The resulting full-length, single-stranded (ss) cDNA contains the complete 5′ end of the mRNA, as well as sequences that are complementary to the SMARTer Oligonucleotide. Amplify sscDNA by LD PCR and get enough dscDNA for library construction.

cDNA was fragmented by dsDNA Fragmentase (NEB, M0348S) by incubate at 37 °C for 30 min. Library construction begins with fragmented cDNA. Blunt-end DNA fragments are generated using a combination of fill-in reactions and exonuclease activity, and size selection is performed with provided sample purification beads. An A-base is then added to the blunt ends of each strand, indexed Y adapters are ligated to the fragments, and the ligated products are amplified with PCR. And then we performed the paired-end sequencing on an Illumina NovaseqTM 6000 at the (LC Sceiences, USA) following the vendor’s recommended protocol.

Cutadapt software was used to remove the reads that contained adapter contamination. And after removed the low-quality bases and undetermined bases, we used HISAT2 software to map reads to the genome. The mapped reads of each sample were assembled using StringTie. The expression levels of all transcripts were estimated and performed as calculating FPKM. The differentially expressed mRNAs were selected with fold change ≥2 or fold change <0.5 and *p* value < 0.05 by edgeR.

### Target prediction of identified DEmiRNAs

High-confidence target genes of the DEmiRNAs were predicted by taking the intersection of results from TargetScan 7.0 (context score percentile ≥50) and miRanda (Δ*G* ≤ −10 kcal mol⁻¹, score ≥140), integrated in the ACGT101-miR pipeline (LC-Bio, Hangzhou, China). We considered only the unique targets of upregulated and downregulated miRNAs for further analysis, excluding genes targeted by both types of DEmiRNAs. The predicted targets were compared to the identified DEGs from our study, and overlapping genes with an opposite trend in expression compared to its associated DEmiRNA were selected; considered targets of upregulated miRNAs should be downregulated in expression and vice versa.

To identify bona fide targets of the secreted and retained miRNAs, we queried the multiMiR R package (v.1.24.0)^51^ for entries annotated as “functional MTI” (weak included) and experimentally supported by either luciferase reporter assays or CLIP experiments, thereby retaining only luciferase- or CLIP-validated miRNA–target interactions for subsequent functional analyses.

### Functional analysis and disease association analysis

Gene Ontology (GO) term enrichment analysis was performed using the clusterProfiler R package (v4.3.2). Categories with false discovery rate (FDR) < 0.05 (*p* < 0.05) were considered as significantly annotated. KEGG pathway enrichment analysis of the DEGs was done by the DAVID (https://david.ncifcrf.gov/) database, with *p* < 0.05 considered as statistically significant. To explore the biological functions of the identified miRNAs, we first mapped their high-confidence target genes and then queried the GeneAnalytics suite^44^ for associated human diseases. Diseases were ranked by GeneAnalytics score, and the top 20 were screened for items of interest. Disease–gene and disease–gene–miRNA regulatory networks for the selected disorders were constructed and visualized in Cytoscape.

### Protein-protein interaction (PPI) network for DEmiRNA-targeted DEGs

Based on the cytoskeleton- and cell-cycle–related KEGG pathways identified among the intracellular DEmiRNA-targeted DEGs, protein–protein interaction networks were constructed with STRING v12.0 (https://string-db.org). The resulting interaction tables were imported into Cytoscape 3.10.0 for network layout and visualization. To identify key regulators in the PPI network, we used the CytoHubba plugin in Cytoscape to rank proteins based on their Matthews Correlation Coefficient (MCC)^52^. The top 10 genes with the highest MCC scores were selected as hub genes for further analysis.

### Statistical analysis

Statistics were derived using GraphPad Prism (v8.0.2) or R (version 4.3.2). The statistical significance was assessed by two-tailed t-test or Fisher’s exact test as indicated in the figure legends. A *p* value less than 0.05 was considered significant.

## Supplementary information

Supplementary Information

Data S1

Data S2

Data S3

Data S4

Data S5

Data S6

Data S7

## Data Availability

Data supporting the findings of this study are available within the article and its Supplementary information files or from the corresponding author upon reasonable request.
