# Supplementary material for: Comprehensive analysis of miRNA expression, secretion profile, and functional network during myogenesis in response to spaceflight
Source: NPJ Microgravity. 2026 Jul 11;12:93. doi: 10.1038/s41526-026-00632-x (PMC13728116; doi:10.1038/s41526-026-00632-x)
Supplement: Supplementary file 1 [file 41526_2026_632_MOESM1_ESM.docx]

Supplementary Information

**Comprehensive analysis of miRNA expression, secretion profile, and functional network during myogenesis in response to spaceflight**

Wenqing Kong^1,2#^, Mei Ma^1,2#^, Zhuoyang Li^1^, Linjun Wang^4^, Ruisen Ma^1^, Siyi Shen^1,2^, Xiaojun Yan^4^, Ping Fan^4^, Zhoumin Niu^1^, Jingjing Chen^1^, Tianyuan Qiu^1^, Nuo Shi^1^, Yuting Wu^1^, Tao Zhang^4^, Yuying Li^1,2*^, Hao Ying^1,2,3*^

^1^CAS Key Laboratory of Nutrition, Metabolism and Food Safety, Shanghai Institute of Nutrition and Health, University of Chinese Academy of Sciences, Chinese Academy of Sciences, and Shanghai Jiao Tong University Affiliated Sixth People's Hospital, Shanghai, China;

^2^Innovation Center for Intervention of Chronic Disease and Promotion of Health;

^3^Key Laboratory of Food Safety Risk Assessment, Ministry of Health, Beijing 100021, China.

^4^Shanghai Institute of Technical Physics of the Chinese Academy of Sciences, Shanghai, 200083, China.

^#^These authors contributed equally to this work.

^*^To whom correspondence may be addressed.

Yuying Li, liyuying@sinh.ac.cn, 86-021-54920960

Hao Ying, yinghao@sinh.ac.cn, 86-021-54920247

The authors declare no conflict of interest.

**Keywords:**

Space flight, Skeletal muscle, miRNA, Secretion, RNAseq

**Figure S1** **Functional effects of dysregulated miRNAs during spaceflight on C2C12 cells, related to Figure 4.**

A-B. FPKM values of target genes regulated by miRNAs that were upregulated (A) or downregulated (B) under spaceflight conditions, as determined by Smart-Seq.

C. qPCR analysis of miRNA expression in C2C12 cells transfected with miRNA mimics or negative control RNA (NC).

D. qPCR analysis of potential target gene Ccna2 expression in C2C12 cells transfected with miR-150-5p mimics or NC.

E. IF staining for MyHC on differentiated C2C12 after 2 days. Scale bar, 100 μm.

F. Quantification of the percentage of MyHC^+^ cells relative to total cells in (E).

G. qPCR analysis of fusion-related gene expression in C2C12 cells transfected with miRNA mimics or NC.

For (C, D F, and G), data represent mean ± SD. Statistical analysis was performed by t test (*p < 0.05; **p < 0.01; ***p < 0.001; ns, not significant).

**Figure S2** **Modulation of miRNA secretion by RBPs, related to Figure 5.**

A. qPCR analysis of Hnrnpa2b1 expression in C2C12 cells transfected with siRNA targeting Hnrnpa2b1 or NC.

B. qPCR analysis of miR-501-3p expression in C2C12 cells and culture medium transfected with siRNA targeting Hnrnpa2b1 or NC.

C. Western blot analysis of HNRNPA1 after immunoprecipitation with HNRNPA1 antibody.

For (A and B), data represent mean ± SD. Statistical analysis was performed by t test (**p < 0.01; ***p < 0.001; ns, not significant).

**Figure S3 Uncropped and unprocessed scans of blots from the figures.**

A. Uncropped and unprocessed scans of blots of Figure 5G.

B. Uncropped and unprocessed scans of blots of Figure S2C.

**Table S1 Primer information.**

Primer sequences used for RT-qPCR.

| Name | Sequence（5’-3’） |
| --- | --- |
| Clip4-F | GAATCCACACTGACATTGCCTCC |
| Clip4-R | TCTTCGGGTAGCTCTGCTTGTG |
| Zmat3-F | AGAACCATGCCAAGAGACTCCG |
| Zmat3-R | TCACTCCCTTCTTTCCGAGTCC |
| Maoa-F | GGCTGTCATCAAGTGCATGGTG |
| Maoa-R | GCAGGCATTGACCCATCTGGTT |
| Slc8a1-R | GCCACAAAATACACAGTTGCTCT |
| Slc8a1-F | AACCACTGAATGTACTGGCTCAT |
| Tfrc-F | GAAGTCCAGTGTGGGAACAGGT |
| Tfrc-R | CAACCACTCAGTGGCACCAACA |
| Cdk1-F | CATGGACCTCAAGAAGTACCTGG |
| Cdk1-R | CAAGTCTCTGTGAAGAACTCGCC |
| Ccnb1-F | AGAGGTGGAACTTGCTGAGCCT |
| Ccnb1-R | GCACATCCAGATGTTTCCATCGG |
| Ccna2-F | TTGTAGGCACGGCTGCTATGCT |
| Ccna2-R | GGTGCTCCATTCTCAGAACCTG |
| Gapdh-F | AGGTCGGTGTGAACGGATTTG |
| Gapdh-R | TGTAGACCATGTAGTTGAGGTCA |
| mmu-miR-150-5p-F | TCTCCCAACCCTTGTACCAGTG |
| mmu-miR-344d-3p-F | CGATATAACCACTGCCAGACTGA |
| mmu-miR-381-3p-F | CTATACAAGGGCAAGCTCTCTGT |
| mmu-miR-143-3p-F | GGTGAGATGAAGCACTGTAGCT |
| mmu-miR-143-5p-F | GCGGTGCAGTGCTGCAT |
| mmu-miR-210-3p-F | CTGTGCGTGTGACAGCGG |
| mmu-miR-501-3p-F | CCACCCGGGCAAGGATTT |
| U6-F | GGAACGATACAGAGAAGATTAGC |
| 18s-F | ACCGCAGCTAGGAATAATGGA |
| 18S-R | CAAATGCTTTCGCTCTGGTC |
| Hnrnpa2b1-F | CAGGGTAGTTGAGCCAAAACG |
| Hnrnpa2b1-R | TTCCAGACTGCCTATCGGTAA |
| Hnrnpa1-F | CGAAACAACCGACGAGAGTCTG |
| Hnrnpa1-R | CATGGCAGCATCCACTTCTTCC |

**Data S1.** **Differentially expressed miRNAs (DEmiRNAs) in C2C12 cells under space flight culture versus ground control.**

Significance was defined as Fisher’s exact test p < 0.05.

**Data S2. Smartseq-identified differentially expressed candidate target genes of intracellular DEmiRNAs in C2C12 cells used for heatmap in Figure 2B.**

Significance was defined as Fisher’s exact test p < 0.05.

**Data S3. Differentially expressed miRNAs detected in C2C12 cell culture medium under space flight culture versus ground control.**

Significance was defined as Fisher’s exact test p < 0.05.

**Data S4. Co-regulation of miRNAs in C2C12 cells and their conditioned medium under space-flight versus ground control.**

Only miRNAs that were significant (Fisher’s exact test, p < 0.05) in the conditioned medium of C2C12 cells under space-flight versus ground control are listed.

**Data S5. Medium/Cell-enrichment shift of miRNAs in C2C12 cells under space-flight versus ground control.**

A positive Δscore indicates miRNA that was secreted after space flight, while a negative value indicates retained miRNA.

**Data S6. Luciferase- and CLIP-validated target genes of secreted DEmiRNAs and their disease associations.**

The full list of validated target genes was retrieved using the multiMiR R package (v1.24.0). Disease enrichment results were obtained from GeneAnalytics analysis.

**Data S7. Luciferase- and CLIP-validated target genes of retained DEmiRNAs and their disease associations.**

The full list of validated target genes was retrieved using the multiMiR R package (v1.24.0). Disease enrichment results were obtained from GeneAnalytics analysis.
